# Infection pattern and transmission potential of chikungunya virus in two New World laboratory-adapted *Aedes aegypti* strains

**DOI:** 10.1038/srep24729

**Published:** 2016-04-22

**Authors:** Shengzhang Dong, Asher M. Kantor, Jingyi Lin, A. Lorena Passarelli, Rollie J. Clem, Alexander W. E. Franz

**Affiliations:** 1 Department of Veterinary Pathobiology, University of Missouri, Columbia, Missouri, United States of America; 2 Division of Biology, Kansas State University, Manhattan, Kansas, United States of America

## Abstract

Chikungunya virus (CHIKV) is an emerging mosquito-borne virus belonging to the *Togaviridae*, which is transmitted to humans by *Aedes aegypti* and *Ae. albopictus*. We describe the infection pattern of CHIKV in two New World *Ae. aegypti* strains, HWE and ORL. Both mosquito strains were susceptible to the virus but showed different infection patterns in midguts and salivary glands. Even though acquisition of a bloodmeal showed moderate levels of apoptosis in midgut tissue, there was no obvious additional CHIKV-induced apoptosis detectable during midgut infection. Analysis of expression of apoptosis-related genes suggested that CHIKV infection dampens rather than promotes apoptosis in the mosquito midgut. In both mosquito strains, the virus was present in saliva within two days post-oral infection. HWE and ORL mosquitoes exhibited no salivary gland infection barrier; however, only 60% (HWE) to 65% (ORL) of the females had released the virus in their saliva at one week post-oral acquisition, suggesting a salivary gland escape barrier. CHIKV induced an apoptotic response in salivary glands of HWE and ORL mosquitoes, demonstrating that the virus caused pathology in its natural vector.

Chikungunya virus (*Togaviridae*; *Alphavirus*; CHIKV) is a newly emerging arbovirus originating from Africa/South Asia, which caused outbreaks in southern Europe in 2006–2007 and two years ago was reported for the first time in the Caribbean Islands^1^. Since then, the virus has caused severe disease outbreaks in 45 countries and territories throughout the Americas, including Columbia, Venezuela, Brazil, Mexico, and the Caribbean Islands^2^. In 2014, a small outbreak was reported in Florida, USA^3^. In humans, CHIKV causes fever, rash, headache, myalgia, and frequently long-lasting arthritis. Like other alphaviruses, CHIKV is transmitted by mosquitoes. Major vectors are *Aedes aegypti* and *Ae. albopictus* with particular virus strains being more efficiently transmitted to vertebrate hosts by *Ae. albopictus* than by *Ae. aegypti*^4^,^5^,^6^. In both mosquito species, the virus has a short minimal extrinsic incubation period (EIP) of only 2 days during which the virus replicates to high titres^7^,^8^,^9^. In the typical urban disease cycle, the virus circulates directly between humans and mosquitoes.

The short CHIKV EIP and production of high titres in its natural vector, *Ae. aegypti*, prompted us to use this virus as a model for ongoing studies aimed at investigating the mechanism arboviruses employ to disseminate from the midgut to secondary tissues and/or to cross the barrier between the hemocoel and salivary glands^10^,^11^,^12^. It has been hypothesized that arboviruses may induce the activity of certain proteases to help dismantle the basal lamina surrounding the midgut before being able to disseminate from this tissue^13^. A similar mechanism may be required to enter the salivary glands. A potential mechanism allowing an arbovirus to overcome tissue barriers in the mosquito could be apoptosis, which involves protease activity and may be induced by the infecting virus^14^,^15^,^16^.

Apoptosis is a programmed cell death pathway in vertebrates and invertebrates that is necessary during tissue development and regeneration^17^,^18^. Apoptosis can be also a powerful anti-pathogen defence response, enabling infected cells to be eliminated, thereby restricting the cell-to-cell spread of a pathogen^19^,^20^. Arboviruses such as CHIKV cause apoptosis during infection of their mammalian hosts whereas in the mosquito vector, arbovirus infections have been regarded to cause no or only minimal pathology^21^,^22^. However, apoptotic responses or overexpression of apoptosis-related genes have been observed for specific arbovirus-mosquito strain combinations, including cases in which the mosquito strain was refractory to the virus due to the presence of a strong midgut infection barrier^23^,^24^,^25^. One aim of this work was to elucidate whether CHIKV infection induces or modulates an apoptotic response in two highly susceptible laboratory strains of *Ae. aegypti*. The core apoptotic pathway in *Ae. aegypti* involves Inhibitor of Apoptosis (IAP) antagonist proteins such as Michelob X (*Mx*) antagonising the activity of IAP1, which in turn inhibits the initiator caspase Dronc^14^,^15^,^26^. Following an apoptotic stimulus, *Mx* interacts with IAP1, leading to the release of Dronc, which then activates downstream effector caspases including CASPS7 and CASPS8. Both effector caspases are considered important components of the apoptotic pathway in *Ae. aegypti*. The effector caspases CASPS18 and CASPS19, which are homologs of the *Drosophila* caspase Decay, do not appear to be apoptosis-related as their over-expression does not result in apoptosis even though their activity is regulated by IAP1^14^,^27^. It has been suggested that these two caspases may be involved in innate immunity or some other process during arbovirus dissemination from the midgut. O’Neill and co-workers have shown that induction of apoptosis via virus-mediated overexpression of *reaper* (a *Drosophila* IAP antagonist) had detrimental consequences for both the virus and the mosquito^16^. However, transient inhibition of apoptosis by silencing *dronc* negatively affected arbovirus replication and dissemination in the mosquito, suggesting that a certain level of apoptosis or caspase activity may be important for optimal infection^15^.

In this study, we characterized phenotypic traits of two laboratory-adapted mosquito strains, HWE and ORL, in conjunction with vector competence for CHIKV. Infection patterns of the virus in midguts and salivary glands were analysed at early time points of infection *in situ* and by quantifying virus loads. We discuss possible routes of dissemination, such as the tracheal network that CHIKV may use to spread from the midgut to secondary tissues. We also analysed the presence of apoptosis in midgut and salivary gland tissue in conjunction with CHIKV infection. Induction of apoptosis was analysed in midguts by TUNEL assays, detection of activated effector caspases, and expression profiling of apoptotic pathway-related genes, and in salivary glands by TUNEL assays.

## Results

### ORL mosquitoes ingest more blood at a faster rate and produce more eggs than HWE

**Figure 1:**
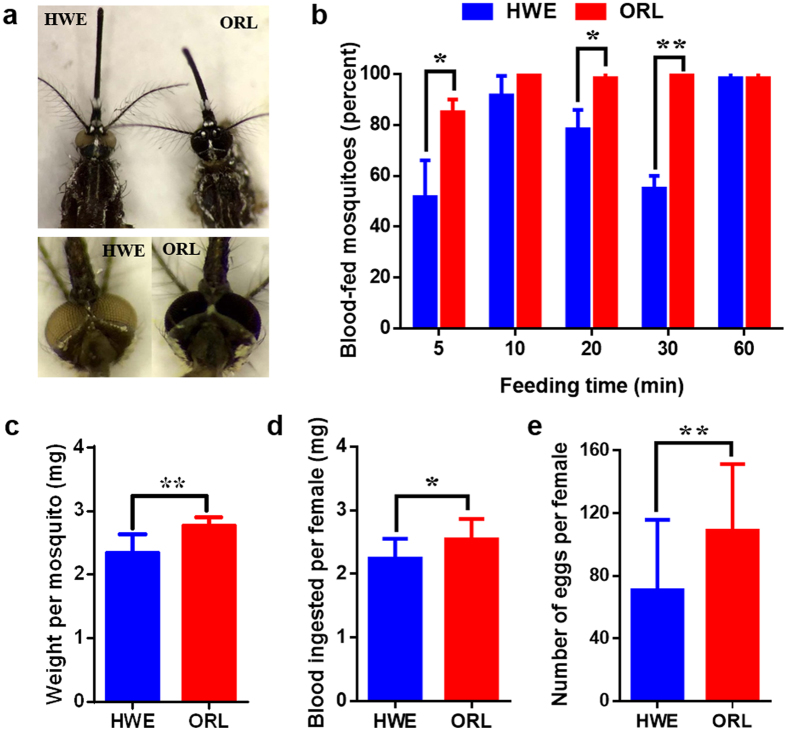
Comparison of feeding behaviour and fecundity between the HWE and ORL strains of *Aedes aegypti.* **(a)** Head and eye regions of HWE and ORL. **(b)** Proportion of HWE and ORL that acquired an artificial bloodmeal during different time intervals (number of individuals per group (n = 20); three replicates). **(c)** Mean body weight of individual (sugarfed) HWE and ORL mosquitoes (n = 60); **(d)** mean blood weight acquired by individual females (n > 200); **(e)** mean fecundity of individual females after a single gonotrophic cycle (n = 30). Data are presented as mean values with SD. Significance was determined by the unpaired two-tailed t-test (* at p ≤ 0.05 and ** at p ≤ 0.01).

The most obvious phenotypic difference between HWE and ORL mosquitoes is the colour of their eyes; HWE mosquitoes have relatively transparent white eyes due to eye-pigment deficiency^28^, whereas ORL mosquitoes have eyes with deep brown colour pigmentation (Fig. 1a). There are additional noticeable phenotypic traits that differ between the two highly laboratory-adapted strains. Blood ingestion efficiency significantly differed between ORL and HWE mosquitoes. Within a 30 min feeding period, significantly more ORL than HWE mosquitoes had ingested blood from artificial bloodmeals with 20/20 ORL females in each experimental replicate being already fully engorged after 10 min. In contrast, 1h feeding time was required for all 20 HWE females of each experimental replicate to feed (Fig. 1b). Based on our observations, it appeared that HWE females needed a longer time period to locate the bloodmeal-containing feeder than ORL. Thus, it is possible that the delay in feeding time may be due to the eye pigment deficiency of HWE, making it harder for the mosquitoes to visually find and recognize the bloodmeal source. ORL females were heavier (Fig. 1c) and ingested a significantly higher amount of blood (an average of 2.54 mg blood per female) than HWE (an average of 2.24 mg blood per female) (Fig. 1d), which could explain why egg production was also significantly higher in ORL than in HWE (Fig. 1e). Typical CHIKV titres in the bloodmeals were around 1 × 10^7^ pfu/ml and 1 μl bloodmeal weighs around 1 mg. Thus, individual ORL and HWE females ingested on average 25,450 and 22,420 infectious CHIK virions, respectively.

### Midguts of ORL mosquitoes have a lower susceptibility to CHIKV than those of HWE

**Figure 2:**
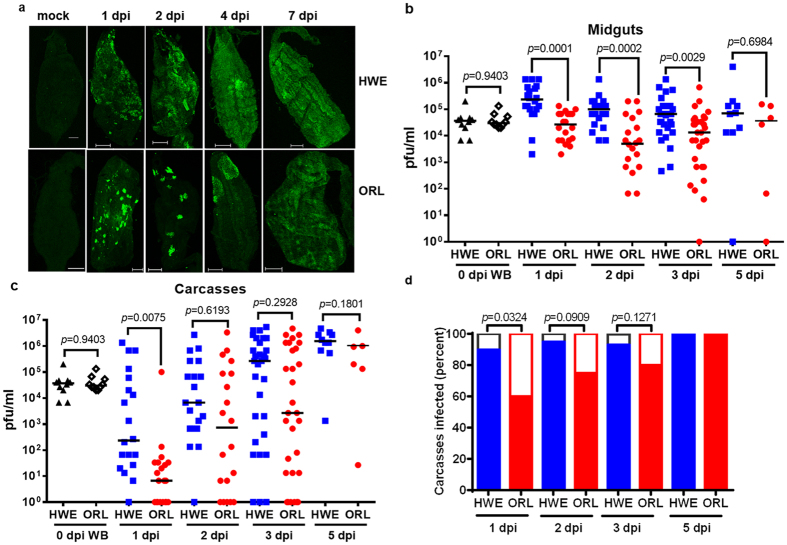
Comparison of CHIKV infection patterns and titres in midguts of HWE and ORL mosquitoes. **(a)** Immunofluorescence assay (IFA) detection of CHIKV-antigen in midguts of HWE and ORL mosquitoes at different time points post-infectious bloodmeal (1, 2, 4, 7 dpi). The mock control consisted of non-infected midguts. Bar = 200 μm. CHIKV titres of individual **(b)** midguts and **(c)** carcasses of HWE and ORL mosquitoes at 0, 1, 2, 3, 5 dpi as analysed by plaque assays in Vero cells. Each data point represents the CHIKV titre of a single midgut or carcass. For 0 dpi, only whole-body (WB) individuals were assayed. Only infected mosquitoes were included in the statistical analysis based on the Mann-Whitney U-test to determine *P* values. Black bars indicate medians. (**d)** Prevalence of CHIKV infection in carcasses of HWE and ORL mosquitoes at 1, 2, 3, 5 dpi. Fisher’s exact test was used to determine *P* values.

Since HWE and ORL mosquitoes showed differences in their bloodfeeding behaviour, we tested whether these two mosquito strains also had different levels of vector competence for CHIKV. In both mosquito strains, midgut tissue showed clear foci of virus infection as early as 1 day post-infection (dpi) (Fig. 2a; Fig. S1). However, the pattern of infection varied between the two mosquito strains with ORL showing small, spotted infection foci at 1–2 dpi, whereas HWE exhibited large, relatively uniform areas of positive staining for viral antigen (Fig. 2a, Figs S2, S3). Median virus titres were significantly lower (~1 log pfu/ml) in midguts of ORL than in HWE at 1, 2 and 3 dpi (Fig. 2b, Table S1), even though 100% of individuals (n = 20) had CHIKV-infected midguts at 1 dpi. Maximal median virus titres were observed at 1 dpi in midguts of HWE and at 5 dpi in those of ORL. In contrast to earlier observations with Sindbis virus (*Togaviridae*; *Alphavirus*; SINV)^29^, we did not observe that CHIKV-infected midgut-associated circular or longitudinal muscles in HWE or ORL mosquitoes (Fig. S3).

**Figure 3:**
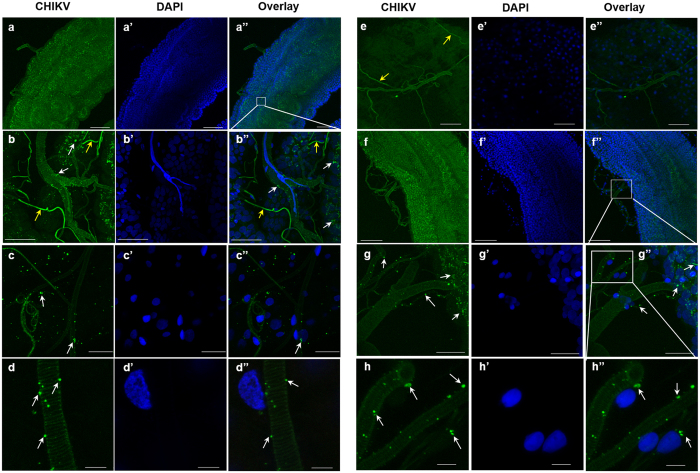
CHIKV infection of midgut-associated tracheal cells in HWE and ORL mosquitoes. **(a–e)** Midguts/tracheae from HWE mosquitoes at **(a,b)** 4 dpi, **(c,d)** 7 dpi, **(e)** 4 days post-bloodmeal (pbm; without CHIKV) and **(f–h)** midguts/tracheae from ORL mosquitoes at 7 dpi were labelled with CHIKV mouse monoclonal antibody (green) to detect viral antigen by IFA. Cell nuclei were stained with DAPI (blue). **(b,g,h)** Images are higher magnification views of **(a,f)**, respectively as outlined by the white boxes. White arrows indicate examples of CHIKV antigen and yellow arrows indicate auto-fluorescence. Bars: **(a,f)** 100 μm, **(b,e)** 50 μm, **(c)** 20 μm, **(d,h)** 5 μm, and **(g)** 25 μm.

At 1 dpi, viral antigen was only faintly visible in tracheal cells of HWE females (Fig. S2; data for ORL not shown) even though CHIKV antigen was abundant in neighbouring midgut epithelial cells. At 4 and 7 dpi, infection patterns looked similar between HWE and ORL with CHIKV antigen being increasingly detected in midgut-associated tracheae of both mosquito strains (Fig. 3).

### CHIKV disseminates from the midguts of infected HWE and ORL mosquitoes within one day post-oral acquisition

In both mosquito strains, CHIKV disseminated from midguts by 1 dpi, even though the dissemination rate was significantly lower for ORL in comparison to HWE (Fig. 2c,d; Fig. S4; Table S1). Until 3 dpi, median CHIKV titres in ORL carcasses were at least 1 log pfu/ml lower in comparison to HWE carcasses. At 5 dpi, virus titres in carcasses of both mosquito strains reached their maximal titres with all mosquitoes showing disseminated infections.

When comparing CHIKV titres of individual mosquito midguts with those of their carcasses, it became obvious that ORL females exhibited a temporary midgut escape barrier to the virus (Fig. S4). Specifically, at 1 dpi, only 60% of ORL females had disseminated CHIKV infections even though 85% of the individual midgut virus titres ranged between 10^4^–10^5^ pfu/ml and did not increase any further during the one-week period (Fig. 2b,c, Fig. S4). Thus, the virus titre in the midgut did not correlate with successful virus dissemination at a given time point, indicating that factors other than virus concentration determine virus escape from the midgut.

### Acquisition of a non-infectious bloodmeal induces apoptosis in midgut tissue

**Figure 4:**
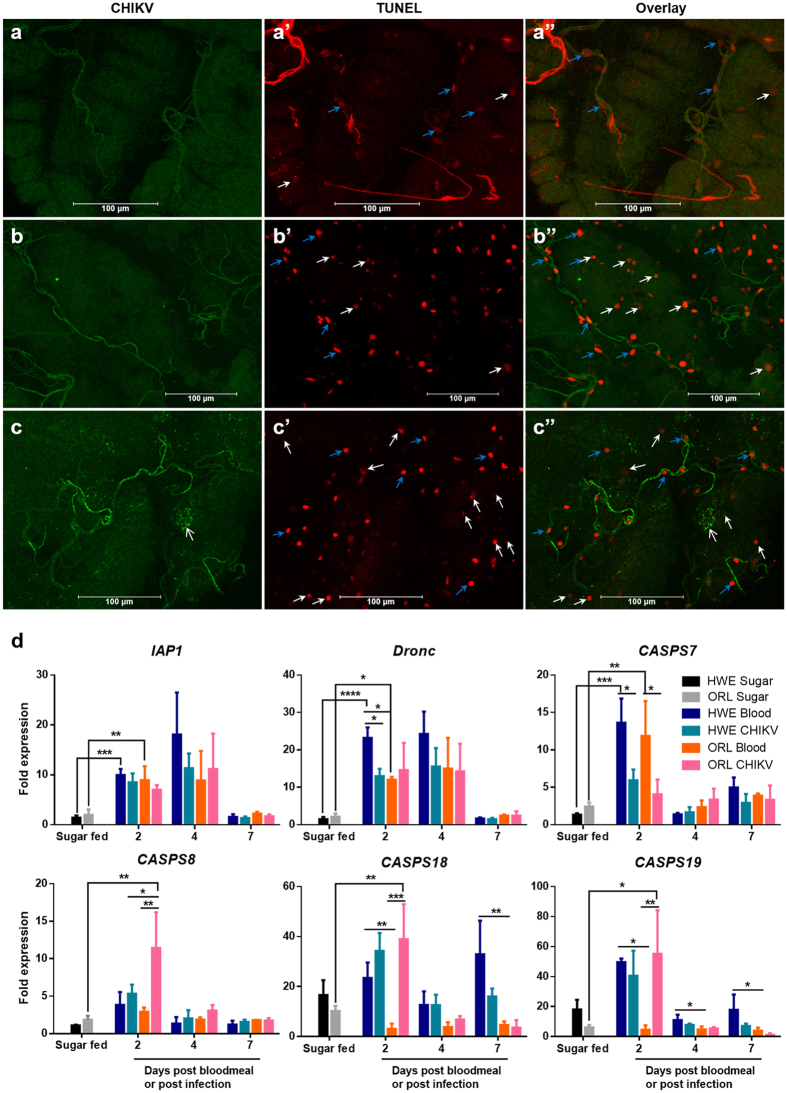
Detection of apoptosis in midguts of bloodfed and CHIKV-infected HWE mosquitoes and relative expression patterns of apoptotic pathway-related genes in HWE and ORL mosquitoes. (**a–c**) Midguts from HWE mosquitoes, which had been fed on (**a**) sugar, (**b**) defibrinated sheep blood mixed with non-infected cell culture medium, or (**c**) defibrinated sheep blood mixed with CHIKV-infected cell culture at 2 days pbm/pi. CHIKV antigen was detected by IFA using a CHIKV-specific mouse monoclonal antibody (green) and apoptosis was detected by TUNEL assay (red). Open white arrows indicate examples of CHIKV antigen, blue arrows indicate examples of TUNEL-positive nuclei of tracheal cells and white arrows indicate TUNEL-positive epithelial cells. Bars: 100 μm. (**d**) Fold-change of transcript abundance of core apoptotic pathway genes and of *casps18* and *casps19* in midguts of bloodfed/CHIKV-fed HWE and ORL mosquitoes. Total RNA was extracted at 2, 4 and 7 days pbm/pi from midguts of HWE and ORL mosquitoes, which had received a sugarmeal, a non-infected cell culture diluted bloodmeal (1:1), or a bloodmeal containing CHIKV-infected cell culture (1:1). Relative gene expression was detected by qRT-PCR and normalised against abundance of ribosomal protein S7 transcripts. Mean values with standard deviation (SD) from three independent experiments are shown. Statistical analysis was performed using one-way analysis of variance (ANOVA) followed by Tukey’s multiple comparisons test (* at p ≤ 0.05).

At 2 days post-bloodmeal/post-infection (pbm/pi), TUNEL-positive, apoptotic nuclei were readily detected in epithelial midgut cells of bloodfed HWE and ORL mosquitoes, regardless of the presence or absence of CHIKV (Fig. 4a–c, Fig. S5). However, the presence of CHIKV did not lead to an obvious increase in the number of apoptotic cells in the midgut epithelium. In midgut cells of sugarfed mosquitoes, TUNEL-positive nuclei were only occasionally visible. In addition, most, if not all nuclei of midgut-associated tracheal cells uniformly and non-specifically stained positive in TUNEL assays, as previously observed^16^. Further analysis of tracheal nuclei at high magnification clarified that those nuclei were not apoptotic as they appeared intact (data not shown).

**Figure 5:**
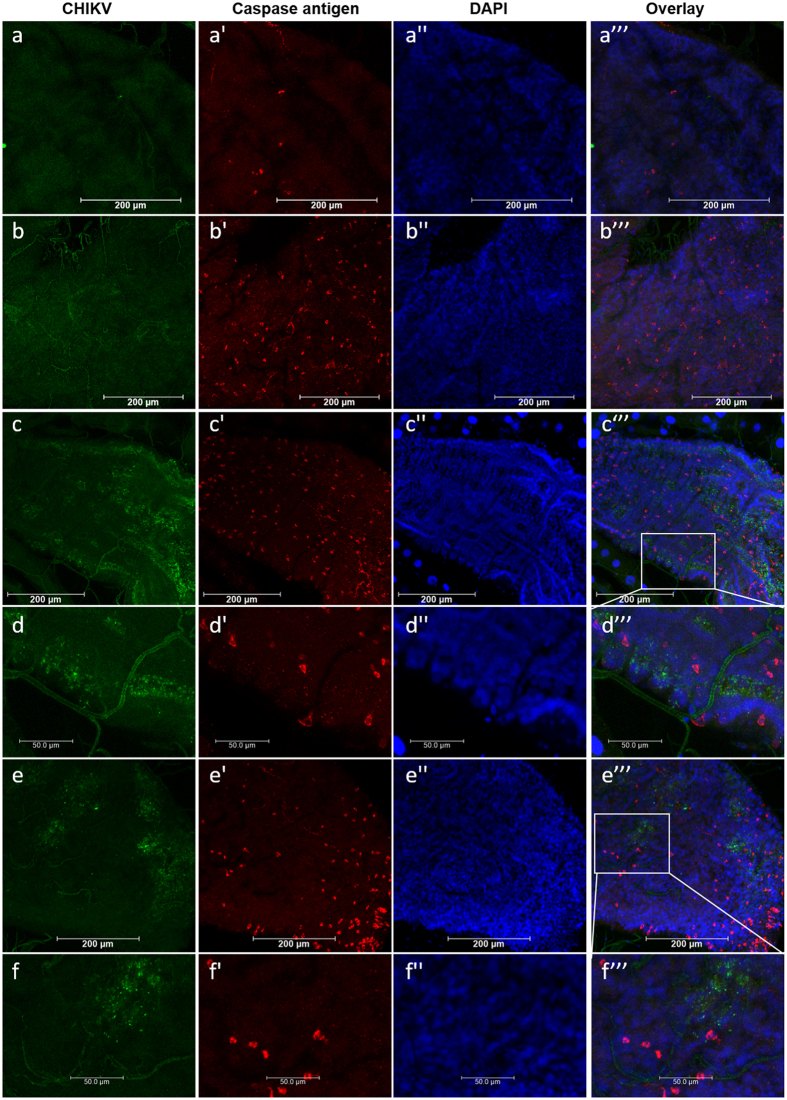
Detection of activated (cleaved) caspase antigen in epithelial cells of bloodfed and CHIKV-infected midguts of HWE and ORL mosquitoes. (**a–d**) Midguts from HWE mosquitoes, which had been fed (**a**) on sugar, (**b**) defibrinated sheep blood mixed with non-infected cell culture, or (**c,d**) defibrinated sheep blood mixed with CHIKV-infected cell culture at 2 days pbm/pi. (**e,f**) Midguts from ORL mosquitoes, which had been fed with defibrinated sheep blood mixed with CHIKV-infected cell culture at 2 dpi. In IFAs, CHIKV antigen was detected by using a CHIKV-specific mouse monoclonal antibody (green); apoptosis was detected by using polyclonal antibodies that detect activated caspase (red), and cell nuclei were stained with DAPI (blue). (**d,f**) Images are higher-magnification views of the area outlined by the white box in (**c,e**), respectively. Bars: (**a–c,e**) 200 μm, (**d,f**) 50 μm.

Using a commercially available antibody raised against activated human caspase-3, which has been shown to be an indicator for apoptosis in *Drosophila*^30^,^31^, we detected activated (cleaved) caspase antigen scattered among midgut epithelial cells of CHIKV or non-infected HWE mosquitoes (Fig. 5). However, activated caspase antigen was not detected in tracheal cells and also did not co-localize with CHIKV antigen. In accordance with the TUNEL assay results, activated caspase antigen was much less abundant in midgut cells of sugarfed mosquitoes compared to those that had received a bloodmeal, further supporting the conclusion that receiving a bloodmeal (without virus) induced apoptosis in midgut tissue.

We analysed relative transcript abundance for the apoptosis-related genes *casps7*, *casps8*, *dronc*, and *iap1* (Fig. 4d). We also included *casps18* and *casps19*, which may be involved in innate immunity or arbovirus dissemination from the midgut^27^. With the exception of *iap1* and *dronc* in midguts of both mosquito strains and *casps18* in HWE midguts, significant changes in gene expression occurred at 2 days pbm/pi, respectively, but less so at later time points. A bloodmeal without virus significantly up-regulated expression of *casps7*, *iap1* and *dronc* in midguts of both mosquito strains at early time points as compared to midguts of sugarfed mosquitoes. In HWE, but not in ORL, *dronc* expression was significantly reduced at 2 dpi in CHIKV-infected midguts in comparison to midguts containing a bloodmeal without virus. *casps7* was significantly down-regulated in CHIKV-infected midguts of both mosquito strains compared to midguts of bloodfed mosquitoes. In midguts of ORL, but not in those of HWE, *casps8*, *casps18*, and *casps19* were significantly upregulated only when CHIKV was present in the bloodmeal. Thus, expression profiles of apoptosis-related and -associated genes were not identical in both mosquito strains.

In summary, we used three different approaches, TUNEL assay, *in situ* detection of activated caspase antigen, and expression profiling of pro-apoptotic genes to determine if CHIKV induces apoptosis during infection of the mosquito midgut. Our results support the conclusion that the presence of a bloodmeal without virus evokes an apoptotic response in midgut tissue of HWE and ORL mosquitoes, whereas the presence of CHIKV in the bloodmeal does not seem to increase the apoptotic response at a detectable level.

### CHIKV infection of salivary glands is accompanied by apoptosis

**Figure 6:**
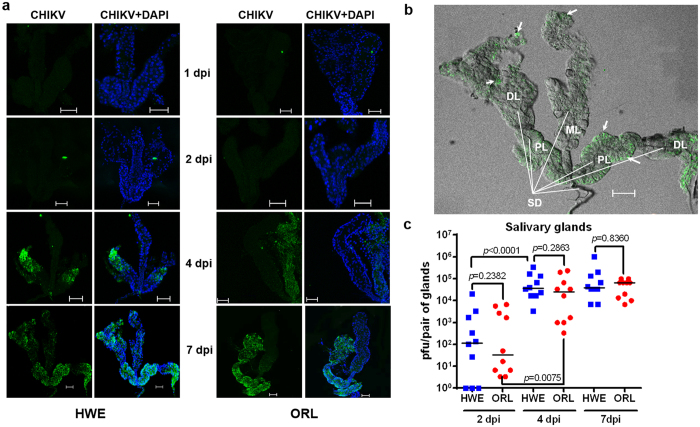
Comparison of CHIKV infection patterns and virus titres in salivary glands of HWE and ORL mosquitoes. **(a)** Salivary glands from HWE (left panels) and ORL (right panels) mosquitoes at 1, 2, 4 and 7 dpi were labelled with CHIKV mouse monoclonal antibody (green) to detect viral antigen by IFA. Nuclei were stained with DAPI (blue). Bars: 50 μm. **(b)** Whole-mount of a salivary gland from a HWE female infected with CHIKV at 7 dpi. The salivary gland is comprised of two lateral lobes and a single, centrally-located medial lobe. **ML:** medial lobe; **PL:** proximal region of lateral lobes; **DL:** distal region of lateral lobes. The main salivary gland duct (**SD)** and ducts of the salivary gland lobes are indicated by white lines. Arrows indicate CHIKV infected areas. Bar: 50 μm. (**c)** Virus titres in individual salivary glands of HWE and ORL mosquitoes at 2, 4, 7 dpi as determined by plaque assay in Vero cells. Each data point represents the CHIKV titre of a single pair of salivary glands. Bars indicate medians.

**Figure 7:**
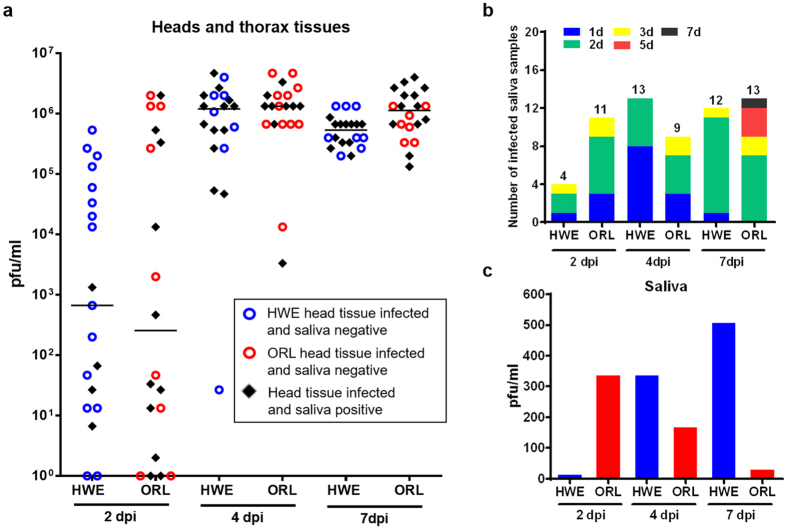
CHIKV presence in saliva and virus titres of saliva. **(a)** CHIKV titres in head and thorax tissues (including salivary glands) from HWE and ORL mosquitoes at 2, 4 and 7 dpi. Virus titres were analysed by plaque assay in Vero cells. Saliva samples were collected from individual females prior to dissecting their head/thorax tissues. Each data point represents the CHIKV titre in a single head/thorax (and saliva) sample. Bars represent medians. (**b)** Number of saliva samples from HWE (n = 20) and ORL (n = 20) mosquitoes collected at 2, 4, and 7 dpi (shown in Fig. 7a) producing CHIKV-typical CPE in inoculated Vero cells. The colour code shows how many saliva samples out of a total number of 20 (per mosquito strain and collection day) caused CPE in Vero cells at 1, 2, 3, 5 and 7 days post-inoculation as an indication for low/high virus titre in a sample. **(c)** Maximal CHIKV titres in saliva samples collected from individual HWE and ORL females at 2, 4, 7 dpi.

Salivary glands are the final key tissue determining transmissibility of CHIKV during its systemic infection of mosquitoes. Our immunofluorescence assay (IFA) data showed that CHIKV infected the salivary glands of both mosquito strains as early as 2 dpi (Fig. 6a). In both mosquito strains, CHIKV was first detected in lateral lobes of the salivary glands at 2–4 dpi, and the virus systemically infected these lobes by 7 dpi. In salivary glands of HWE but not in those of ORL, virus was also detected in the medial lobes at 7 dpi (Fig. 6a,b). Median CHIKV titres in individual salivary glands of HWE reached 100 pfu/glands at 2 dpi and two days later, median virus titres reached their plateau (3.7 × 10^4^ pfu/glands) (Fig. 6c, Table S1). In ORL salivary glands, the median virus titre continuously increased over time, reaching its maximum at 7 dpi, which exceeded that of HWE by almost two-fold. Similar to the weak relationship between midgut virus titres and dissemination efficiency (Fig. S4), salivary glands/head tissue titres did not strongly correlate with virus excretion into saliva, especially at 2 and 4 dpi (Fig. 7a). CHIKV secretion into saliva was confirmed at 2 dpi in 20% of HWE and 55% of ORL females (p = 0.0484, Fisher’s exact test) (Fig. 7, Table S1). Interestingly, even after 7 dpi only 60% (HWE) to 65% (ORL) of the saliva samples contained CHIKV indicating that both mosquito strains had a salivary gland escape barrier for the virus. Mosquitoes that released CHIKV in their saliva showed saliva virus titres that did not exceed 500 pfu/ml at 7 dpi (HWE).

**Figure 8:**
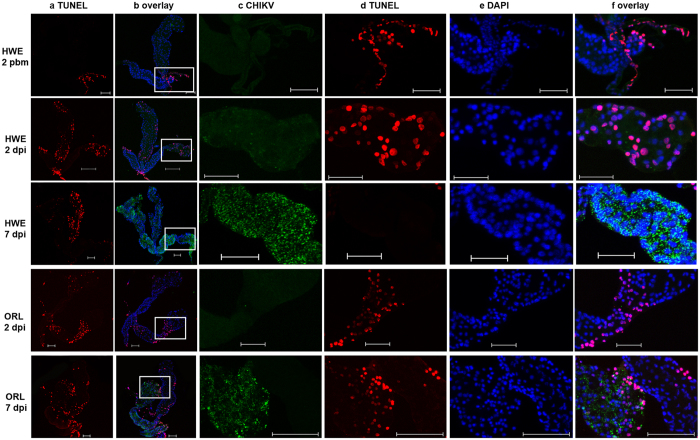
CHIKV induced apoptosis in salivary glands of HWE and ORL mosquitoes. In IFA, salivary glands of HWE (first, second and third rows) and ORL (fourth and fifth rows) mosquitoes were labelled with CHIKV mouse monoclonal antibody to detect viral antigen (green) at 2 and 7 dpi. Apoptotic cells were detected via TUNEL staining (red) and nuclei were stained using DAPI (blue). Panels in columns 3 to 6 are higher-magnification views of the lateral salivary gland lobes outlined by white boxes in column 2. HWE 2 days pbm: HWE salivary glands at 2 days post-feeding with defibrinated sheep blood diluted 1:1 in non-infected cell culture medium. HWE/ORL 2 dpi and 7 dpi: HWE/ORL salivary glands at 2 and 7 days post-feeding with CHIKV-infected cell culture medium diluted 1:1 in defibrinated sheep blood. Bars: 50 μm.

Between 2–7 dpi, apoptotic cells were clearly detected in the salivary glands of CHIKV-infected individuals of both mosquito strains (Fig. 8)-however, in non-infected salivary glands (at 2 days pbm), apoptosis was restricted to the salivary ducts in repeated observations (n = 5) (Fig. 8, upper row). At 2 dpi, little viral antigen was detected in salivary glands from both strains. At this time point, all three salivary gland lobes in HWE showed the presence of apoptotic cells, whereas in ORL apoptosis was mainly observed in lateral lobes (n = 3). At 7 dpi, however, predominantly the medial lobes of HWE salivary glands were apoptotic in repeated observations (n = 3), in contrast to salivary glands of ORL, which showed strong apoptosis in all three lobes. In summary, CHIKV induced apoptosis in salivary glands of HWE and ORL mosquitoes at early time points of infection; however, the patterns of the apoptotic responses varied between the two mosquito strains.

## Discussion

In this study, we examined the infection pattern of CHIKV in midguts and salivary glands of two laboratory-adapted *Ae. aegypti* strains at early time points of infection. Our work confirms earlier reports showing that at 28 °C the mean EIP of CHIKV in *Ae. aegypti* can be as short as 2 days with virus disseminating from the midgut after 1 dpi^6^,^7^,^8^,^9^. Other mosquito-borne arboviruses for which a mean EIP as short as 2 days has been observed include Venezuelan equine encephalitis virus (*Togaviridae*; *Alphavirus*; VEEV) in *Ae. aegypti* and Rift Valley fever virus (*Bunyaviridae*; *Phlebovirus*; RVFV) in *Culex pipiens*^32^,^33^.

HWE and ORL strains were highly susceptible to CHIKV infection and able to release the virus in their saliva. However, within the first 3 dpi, CHIKV progressed at a significantly slower rate in midguts and secondary tissues of ORL females than in those of HWE. In addition, the viral infection pattern in midguts of ORL mosquitoes was more restricted than that of HWE, indicating that in ORL mosquitoes, CHIKV was confronted with temporary midgut infection and escape barriers. A relative inefficiency of the virus to cross the physical barriers separating midgut lumen from the epithelium and the midgut epithelium from secondary tissues in ORL mosquitoes could account for the delayed tissue infections. Another possibility could be an antiviral immune response such as RNA interference, which may be more efficient in ORL than in HWE^34^,^35^. Testing this hypothesis should be the subject of a separate study. Previously, it was assumed that arboviruses need to reach a certain threshold titre in the midgut before disseminating to secondary tissues^36^,^37^. Our observations indicate that CHIKV dissemination from the midgut is not threshold titre-dependent: several mosquitoes with low virus titres in their midguts had disseminated infections at 1–2 dpi, whereas in other individuals with high midgut virus titres, the virus had not disseminated though all mosquitoes had initially ingested similar quantities of virus. Since CHIKV was able to disseminate from the midgut as early as 1 dpi, it seems possible that the virus only requires a single (or very few additional) replication cycles in the midgut epithelium before disseminating to secondary tissues. Based on this assumption, midgut infection foci that were observed by IFA between 1–7 dpi appeared at a time point when the virus had already spread to secondary tissues. Regardless, our data show that only those individuals with CHIKV infections in their midgut could develop infections in secondary tissues later on, indicating that midgut infection is a prerequisite for secondary tissue infections.

Several studies have shown that arboviruses infect the tracheal network that is associated with the midgut epithelium^10^,^11^,^38^,^39^,^40^. It was then speculated that tracheae may form an escape route for viruses from the midgut on their way to infect secondary tissues^11^,^38^,^40^. We observed efficient dissemination of CHIKV from midguts of HWE and ORL as early as 1 dpi. This was a time point at which viral antigen was abundantly present in midgut epithelial cells but was hardly detectable in midgut-associated tracheal cells. Though tracheal cells became increasingly infected with the virus over time, our observations do not confirm that midgut-associated tracheae form the major route of CHIKV dissemination from the midgut. If CHIKV disseminated predominately via the tracheae, one would expect a stronger virus presence in tracheal cells at early time points of infection when dissemination to secondary tissues was already strong.

In salivary glands and excreted saliva, CHIKV was detected in both mosquito strains as early as 2 dpi with maximal virus titres in salivary glands (and mosquitoes as a whole) after 4 dpi. In addition to differences in their midgut infection patterns, both mosquito strains also differed in their salivary gland infection patterns. In HWE, CHIKV first infected the lateral lobes responsible for the production of enzymes required during bloodfeeding and for the release of sugar-digesting enzymes, followed by infection of the medial lobe at 7 dpi whose function is associated with bloodfeeding^41^. Thus, in HWE salivary glands, the CHIKV infection pattern resembled that of DENV2 in *Ae. aegypti*^40^. In contrast, the CHIKV infection pattern of ORL salivary glands, in which the medial lobe did not contain detectable viral antigen, was similar to that of SINV in *Ae. aegypti*^42^,^43^. Around one third of the mosquitoes of both strains did not contain detectable quantities of CHIKV in their saliva at 7 dpi, even though 100% of salivary glands were infected with median virus titres in ORL salivary glands being 1.7x higher than in those of HWE. Thus, both mosquito strains exhibited a salivary gland escape barrier for CHIKV, which did not seem to strongly correlate with the virus concentrations in the infected salivary glands. Instead, it may be possible that the level of apoptosis observed in CHIKV-infected salivary glands (and discussed below) could be an important factor affecting the salivary gland escape barrier in HWE and ORL mosquitoes. So far, salivary gland escape barriers in mosquitoes have been reported for La Crosse virus (*Bunyaviridae*; *Orthobunyavirus*; LACV), SINV, and RVFV^44^,^45^,^46^,^47^,^48^.

In TUNEL assays, apoptotic cells were detected at 2 days pbm/pi in the midgut epithelium of HWE and ORL females, which had received a non-infectious bloodmeal or a bloodmeal containing CHIKV. Expression profiling of apoptotic pathway genes revealed that bloodfeeding alone caused a significant upregulation of *iap1*, *dronc*, and *casps7* in both mosquito strains, which is in line with TUNEL assays and activated caspase detection results. Interestingly, the presence of CHIKV in midgut tissue of both mosquito strains caused a significant reduction of *casps7* expression levels, suggesting that CHIKV is dampening apoptosis rather than inducing and promoting it. These observations support the conclusion that receiving a bloodmeal evokes a moderate apoptotic response in the midgut epithelium either induced by components of the bloodmeal or by the physical stretching of the midgut tissue^49^. Our data do not support the hypothesis that CHIKV induces apoptosis in the midguts of these two susceptible mosquito strains. However, it cannot be ruled out that the apoptotic response caused by the bloodmeal may be beneficial for the virus, for example by promoting its dissemination from the midgut. This could occur if the apoptotic response is involved in midgut epithelial basal lamina remodeling^13^,^49^ and could explain the observation by Wang and colleagues showing that apoptosis in the midgut may be necessary for efficient arbovirus infection and dissemination^15^. *casps18* and *casps19* were significantly upregulated in CHIKV-infected midguts of ORL mosquitoes, whereas in sugar- and bloodfed (without CHIKV) HWE midguts, expression levels for both genes were already elevated and did not increase any further in the presence of virus. Our results confirm earlier observations by showing that both *casps18* and *casps19* share similar expression profiles in similar tissues because *casps18,* lacking its own catalytic domain, is considered to be a decoy caspase acting as an enhancer for *casps19*^27^. At this point, however, the expression profiles of *casps18* and *casps19* in both mosquito strains in relation to immunity or CHIKV infection/dissemination are difficult to explain and require further analysis. In non-infected salivary glands of both mosquito strains, apoptosis was restricted to the saliva ducts of the glands, whereas only in CHIKV-infected salivary glands, apoptotic cells were also detected in the lobes of glands between 2–7 dpi. This clearly shows that a bloodmeal-acquired CHIKV infection causes pathology in its natural mosquito vector, *Ae. aegypti*. Girard and colleagues observed apoptotic cells and tissue in salivary glands of West Nile virus (*Flaviviridae*, *Flavivirus*; WNV)-infected *Cx. quinquefasciatus* females between 7 and 28 dpi^24^,^50^. Also, in non-infected salivary glands, a small number of apoptotic cells was detected, which increased over time. Thus, the authors speculated that even though WNV did induce apoptosis in mosquito salivary glands, the majority of the apoptotic response was due to aging of the mosquito. These observations differ from those of our study, since we observed CHIKV-induced apoptosis in salivary glands at 2 dpi. Instead, our observations are largely in agreement with those made when analysing apoptosis in salivary glands of *Ae. albopictus* and *Ae. aegypti*, which had been intrathoracically injected with SINV or fed with SINV-containing bloodmeals, respectively^42^,^43^. Salivary ducts of SINV-infected and non-infected salivary glands stained positive in TUNEL assays. Further, in SINV-infected salivary glands, apoptotic cells were observed throughout the proximal and distal regions of the lateral lobes at 5–7 dpi. In our study, however, CHIKV also infected the medial salivary gland lobes (in HWE mosquitoes) and induced apoptosis in this tissue. It has been suggested that apoptosis in the salivary glands may be due to cellular homeostasis as well as antiviral defence and may affect virus concentrations in saliva^42^,^51^,^52^. As apoptosis was detected in CHIKV-infected salivary gland tissue but not in uninfected salivary glands (apart from the saliva ducts), it remains to be investigated whether CHIKV-induced apoptosis quantitatively affects the release of virus along with mosquito saliva.

## Material and Methods

### Mosquitoes

Two highly laboratory-adapted *Ae. aegypti* strains were used in this study: the RexD (Puerto Rico)-derived, eye-pigment deficient Higgs White Eye (HWE) strain and the pigmented eye exhibiting Orlando, Florida (ORL) strain^28^,^53^. Mosquitoes of these two strains were reared at 28 °C with 75–80% relative humidity and a 12 h light/12 h dark cycle in a BSL2 insectary. For colony maintenance, mosquitoes received artificial bloodmeals consisting of defibrinated sheep blood (Colorado Serum Company, Denver, CO).

### Quantitative feeding assay and assessment of fecundity

Each group of twenty female HWE or ORL mosquitoes was kept in a 16 oz. (3.9″ × 3.9″) organdie-covered carton. Mosquitoes were starved for 24 h before receiving artificial bloodmeals for 5, 10, 20, 30, or 60 min. Before (*W*_*b*_) and after bloodfeeding (*W*_*a*_) each mosquito-containing carton was weighed. Then the number of blood-engorged females (*n*) was recorded for each group. Amount of blood taken by a single mosquito was calculated according to the formula [(*Wa* − *Wb*)/*n*]. For each time point, three biological replicates were performed.

Fecundity of the two mosquito strains was assessed by placing each individual blood-engorged mosquito (n = 30) in a separate 16 oz. carton containing an oviposition cup filled with water containing a strip of paper towel. After five days, the number of eggs from a single gonotrophic cycle was recorded for each female.

### CHIKV infection of mosquitoes and virus detection in mosquito tissues

CHIKV strain 37997 (West African genotype; GenBank accession: AY726732.1) from Senegal was propagated in Vero cells in T25 flasks at a multiplicity of infection (m.o.i.) of 0.01 using Minimum Essential Medium Eagle (MEM) complemented with 7% FBS. Virus-containing cell culture media was collected at 24–30 h pi and mixed with defibrinated sheep blood at a 1:1 ratio with 10 mM ATP. One week-old females that had been deprived of sugar for 24 h were fed for 1 h with virus-infected cell culture-blood mixture or non-infected cell culture-blood mixture (control) at 37 °C using a single glass feeder per carton. Fully engorged females were reared in 1.9 L (64 oz.) cartons and offered raisins and water until further analysis.

Virus titres from individual mosquitoes, midguts, carcasses, salivary glands and head tissues including thorax were determined by plaque assays. Tissue samples and salivary glands from individual mosquitoes were homogenised in 1 ml and 0.5 ml sample processing buffer (MEM with 7% FBS and 1% HEPES), respectively. Homogenised samples were sterile-filtered using Acrodisc HT Tuffryn 0.2 μm syringe filters (Pall Life Sciences, East Hills, NY). Vero cells were seeded onto 24-well plates and incubated for three days at 37 °C to achieve 95–100% confluence. Cells were infected with 10-fold serial dilutions of each mosquito homogenate. Cells were incubated for 1 h at 37 °C before being overlaid with an agarose nutrient mixture [1x Medium 199 (Sigma-Aldrich, St. Louis, MO, USA), 10% FBS, 4% NaHCO_3_, 0.5% MEM vitamins, 0.5% MEM amino acids (Mediatech Inc., Manassas, VA, USA)]. After incubation at 37 °C for 2 days, cells were stained with MTT (3-[4,5-dimethylthiazol-2-yl]-2,5-diphnyltetrazolium bromide) (Sigma-Aldrich, St. Louis, MO, USA) and incubated at 37 °C for 24 h, and the number of plaques counted for each sample. Viral titres of individual mosquitoes were calculated as plaque forming units per ml (pfu/ml).

All mosquito infections and CHIKV detection assays were carried out in a Biosafety Level 3 laboratory within the Laboratory for Infectious Disease Research (LIDR) of the University of Missouri.

### Saliva collection and analysis for the presence of CHIKV

Saliva was collected from individual mosquitoes in groups of twenty at 2, 4, and 7 dpi using a forced salivation method^54^,^55^. A day before saliva collection, mosquitoes were deprived of sugar. Females were anesthetized by cold treatment and their wings and legs were removed. Proboscises of mosquitoes were inserted into 1 mm glass capillary tubes filled with 3–5 μl Cargille Type B immersion oil. After 1 h, saliva was collected from those capillaries in which droplets of saliva exuding from the proboscis were visible. Saliva samples were recovered from each capillary tube by centrifugation at 3,000 g for 15 min in a 1.5 ml Eppendorf tube containing 200 μl sample processing buffer. After addition of another 200 μl of sample processing buffer, each sample was vortexed and sterile-filtered using a 0.2 μm syringe filter (Pall Life Sciences, East Hills, NY, USA). To amplify virus from saliva samples, Vero cells seeded onto 24-well plates were inoculated with 180 μl saliva samples for 1 h at 37 °C before adding 1 ml of DMEM supplemented with 7% FBS. Cells were observed daily for the development of cytopathic effects (CPE) under an inverted microscope until 7 dpi. Virus titres from infected saliva samples were analysed by plaque assay as described above.

### qRT-PCR

Mosquito midguts were dissected from HWE and ORL females at 2, 4 and 7 days after receiving a CHIKV-containing or a non-infectious bloodmeal, and also from sugarfed mosquitoes. Groups of six midguts were collected for total RNA extraction using Trizol reagent (Invitrogen, Carlsbad, CA, USA) according to the instructions of the manufacturer. First-strand cDNA was synthesised from 1 μg total RNA using QuantiTect Reverse Transcription Kit (Qiagen, Hilden, Germany). Gene specific primers (Table S2) were used for qPCR amplification of apoptosis-associated genes. qPCR amplification and analysis were carried out using the Applied Biosystems (ABI) 7300 Real-Time PCR System. The final reaction volume was 20 μl using ABI SYBR green Supermix (Applied Biosystems, Warrington, UK). The PCR program was: hold at 95 °C for 10 min, 95 °C for 15 seconds and 60 °C for 1 min, repeated 40 cycles. The specificity of the SYBR green PCR signal was further confirmed by a melting curve analysis and agarose gel electrophoresis. The relative abundance of apoptosis-related gene transcripts was normalised and calculated against that of the ribosomal protein S7 gene (AAEL009496) as an endogenous reference using the 2^−ΔΔ^C_T_ method^56^. Each sample had three independent biological replicates.

### Immunofluorescence assays and TUNEL staining

Midguts and salivary glands were dissected from HWE and ORL females at 2, 4 and 7 days pbm/pi. A group of five midguts or 10 salivary glands were fixed in a 1.5 ml Eppendorf tube containing 200 μl 4% p-formaldehyde (Sigma) for up to one week at 4 °C. After three washes with PBS, samples were permeabilised by incubation with 200 μl PBT (1 × PBS, 1% BSA, 0.2% Triton X-100) for 1 h on a rocker at room temperature. Samples were then incubated overnight at 4 °C in 100 μl anti-CHIKV mouse monoclonal antibody [B1414] (Abcam, Cambridge, MA, USA) at a dilution of 1:100 in PBT. A number of midguts were co-incubated with rabbit anti-(cleaved) human caspase-3 antibody (Cell Signaling Technology, Danvers, MA, USA) diluted 1:500 to detect the presence of activated caspase antigen^30^,^31^. After washing four times with 200 μl PBST (0.1% Triton X-100), samples were incubated for 1.5 h with 100 μl 1:200 anti-mouse IgG Alexa Fluor 488 (Cell Signaling Technology) and 1:800 anti-rabbit Alexa Fluor 594 (Cell Signaling Technology) in PBT at 37 °C in the dark. For nuclei staining, 0.5 μl of 0.5 μg/μl DAPI (Invitrogen, Carlsbad, CA, USA) was added to each tube and incubated for another 30 min. For TUNEL staining, secondary antibody solutions of each tube were replaced by 50 μl TUNEL TMR red reaction mixture (Roche Applied Science, Indianapolis, IN, USA) and incubated at 37 °C in the dark for another 1 h. After washing four times with PBST, midgut and salivary gland samples were placed individually on glass slides, mounted with Fluoromount-G (Electron Microscopy Sciences Hatfield, PA, USA), before being covered with cover slips. Samples were viewed and analysed using z-axis function under an inverted spectral confocal microscope (TCP SP8 MP, Leica Microsystems) located at the Molecular Cytology Core of the University of Missouri.

### Data analysis

Statistical analysis was performed using the GraphPad Prism software package (version 6.01). CHIKV titres were compared between HWE and ORL mosquitoes using the non-parametric Mann-Whitney U-test. Prevalence of CHIKV infection in mosquito tissues was analysed using Fisher’s exact test. Differences in mosquito feeding time, amounts of blood taken by individual mosquitoes and oviposition counts were analysed using Student’s T-test. In the qRT-PCR experiment, data from each sample were analysed using one-way analysis of variance (ANOVA) followed by Tukey’s multiple comparisons test. All tests were considered significant at p ≤ 0.05.

## Additional Information

**How to cite this article**: Dong, S. *et al.* Infection pattern and transmission potential of chikungunya virus in two New World laboratory-adapted *Aedes aegypti* strains. *Sci. Rep.* **6**, 24729; doi: 10.1038/srep24729 (2016).

## Supplementary Material

Supplementary Information
