# Supplementary material for: Infection pattern and transmission potential of chikungunya virus in two New World laboratory-adapted Aedes aegypti strains
Source: Sci Rep. 2016 Apr 22;6:24729. doi: 10.1038/srep24729 (PMC4840389; doi:10.1038/srep24729)
Supplement: Supplementary Information [file srep24729-s1.doc]

**Supplementary Information**

**Infection pattern and transmission potential of chikungunya virus in two New World laboratory-adapted *Aedes aegypti* strains**

Shengzhang Dong^1^, Asher M. Kantor^1^, Jingyi Lin^1^, A. Lorena Passarelli^2^, Rollie J. Clem^2^, Alexander W.E. Franz^1,3^

^1^Department of Veterinary Pathobiology, University of Missouri, Columbia, Missouri, United States of America

^2^Division of Biology, Kansas State University, Manhattan, Kansas, United States of America

^3^Corresponding Author, Email: franza@missouri.edu


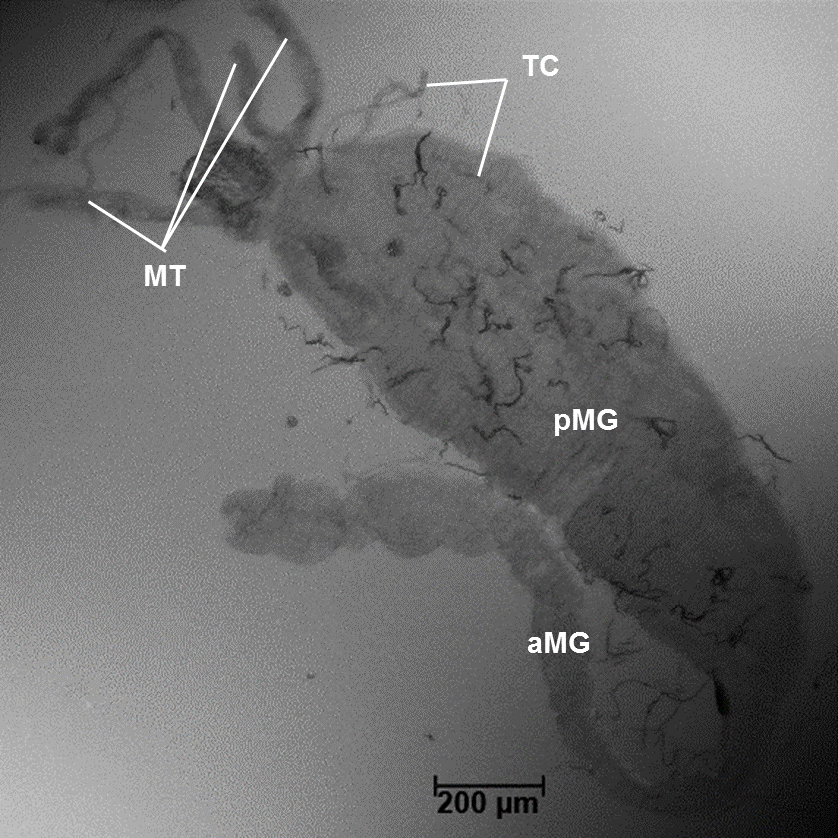


**Figure S1 Bright-field image of a dissected midgut from a HWE female**.

aMG, anterior midgut; pMG, posterior midgut; MT, Malpighian tubules; TC, trachea.


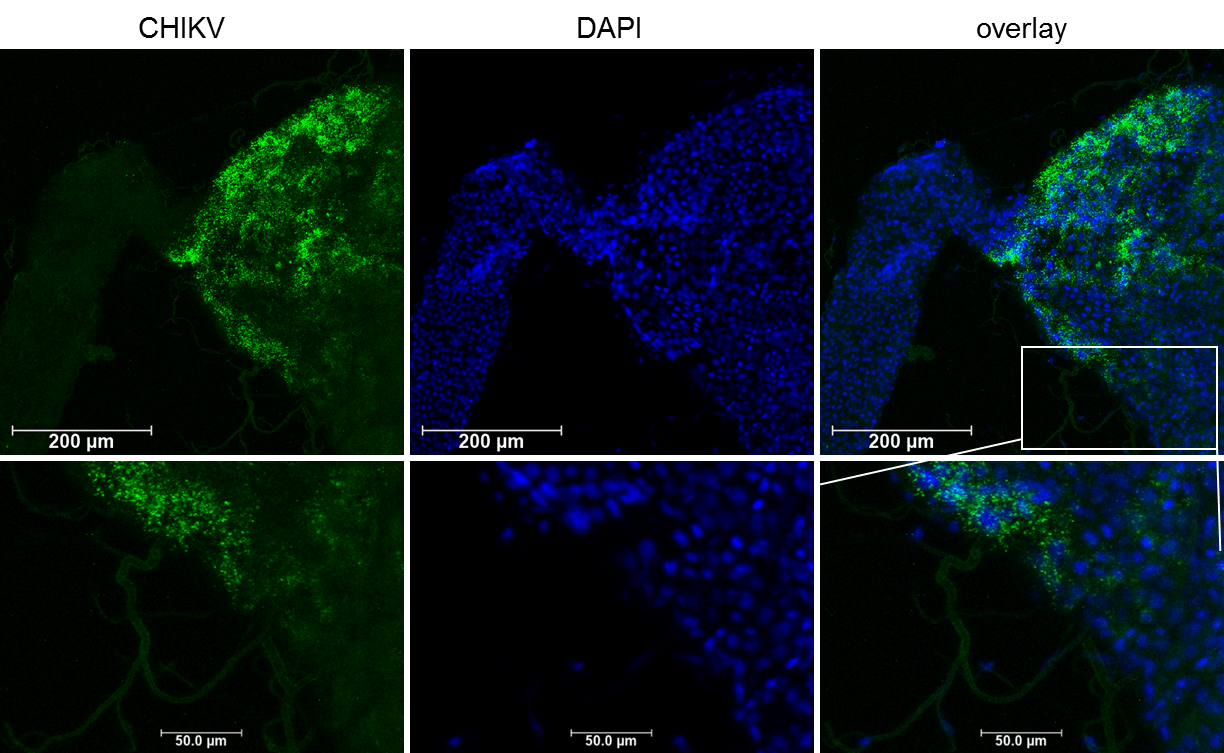


**Figure S2 CHIKV infection in the midgut of a HWE female at 1 dpi – viral antigen is only faintly detectable in tracheal cells.**

In IFA, a midgut from a HWE female was labelled with CHIKV mouse monoclonal antibody (green) to detect viral antigen at 1 dpi. Cell nuclei were stained with DAPI (blue). Images of lower row are magnified views as outlined by the white box. Bars: 200 mm, 50 mm.


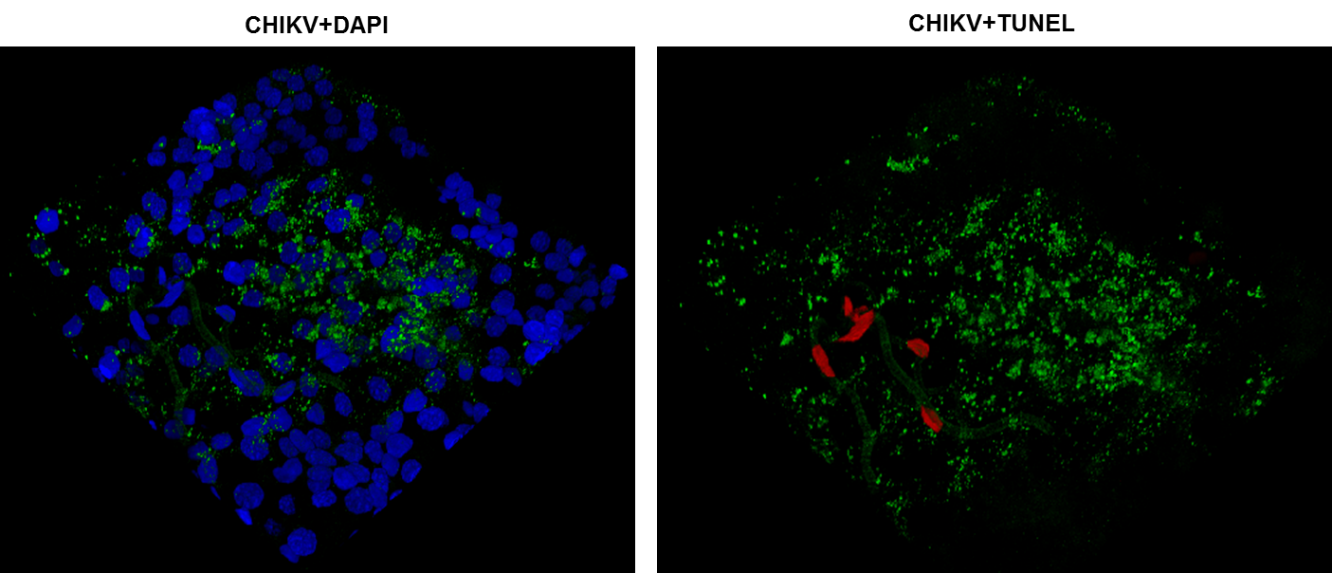


**Figure S3 3-D view of CHIKV infected HWE mosquito midgut at 2 dpi.**

CHIKV antigen was detected in the midgut of a HWE mosquito at 2 dpi by IFA using CHIKV mouse monoclonal antibody (green). Apoptosis was detected by TUNEL assay (red), and cell nuclei were stained with DAPI (blue). Images were captured using a Leica TCP SP8 MP inverted spectral confocal microscope with z-axis function and transferred into 3-D structure using LAS AF 3 software.


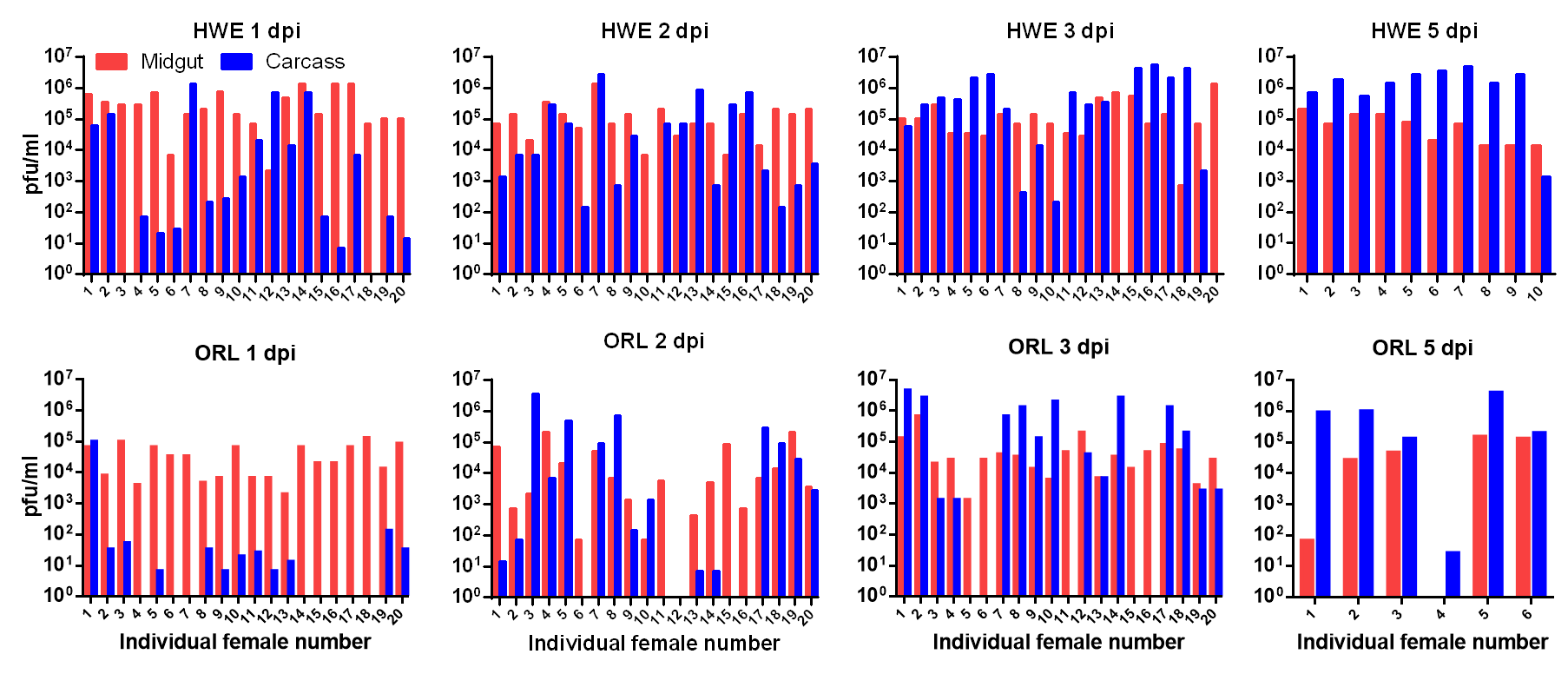


**Figure S4 Comparison of CHIKV titres in midguts (red bars) and carcasses (blue bars) from individual mosquitoes (#1 to #20).**

Midguts and carcasses were dissected from individual HWE (top panels) or ORL (lower panels) mosquitoes at 1, 2, 3, and 5 dpi. Virus titre was determined by plaque assays in Vero cells.


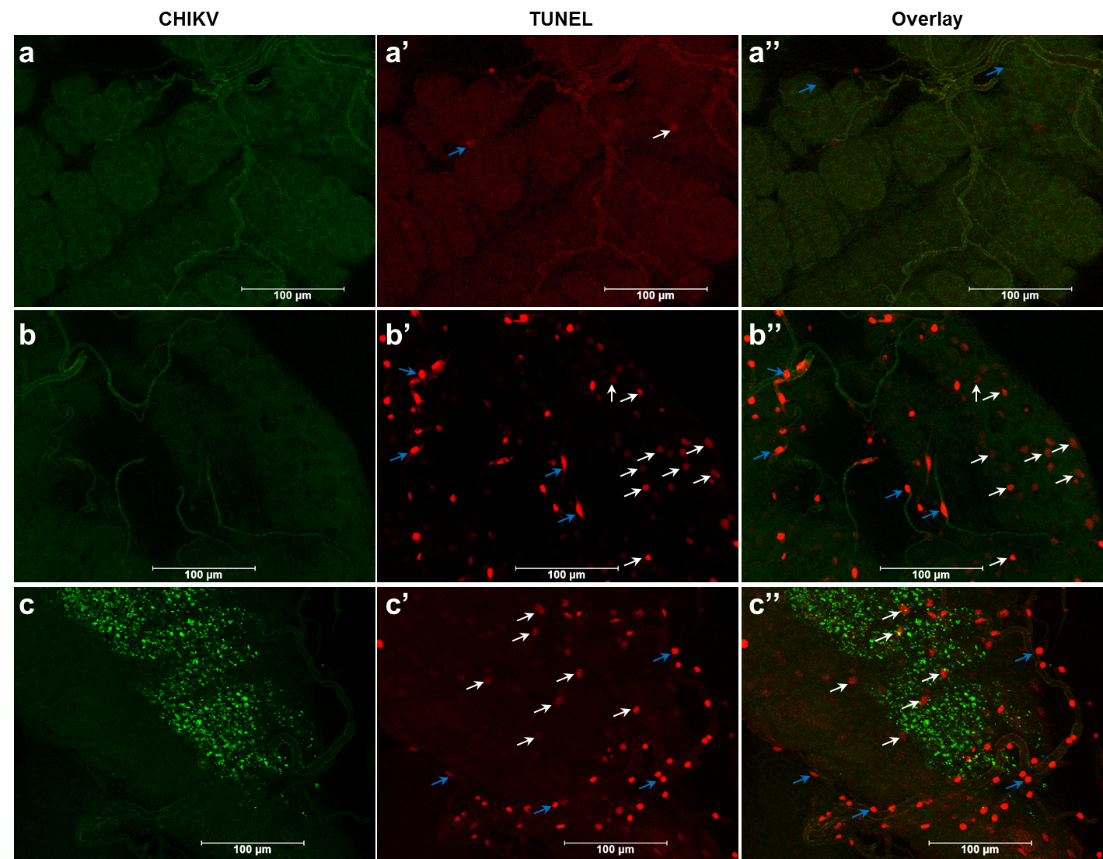


**Figure S5 Detection of apoptosis in midguts of bloodfed (CHIKV-infected) ORL mosquitoes**. **(a)** Midguts from ORL mosquitoes fed on sugar, **(b)** defibrinated sheep blood mixed with non-infected cell culture, and **(c)** defibrinated sheep blood mixed with CHIKV-infected cells at 2 days pbm/pi. CHIKV antigen was detected by IFA using a CHIKV-specific mouse monoclonal antibody (green) and apotosis was detected by TUNEL assay (red). Blue arrows indicate examples of TUNEL-positive nuclei of tracheal cells and white arrows indicate TUNEL-positive epithelial cells. Bars: 100 mm.

**Table S1.** **CHIKV infection dynamics and intensity over time in different tissues of HWE and ORL mosquitoes.**


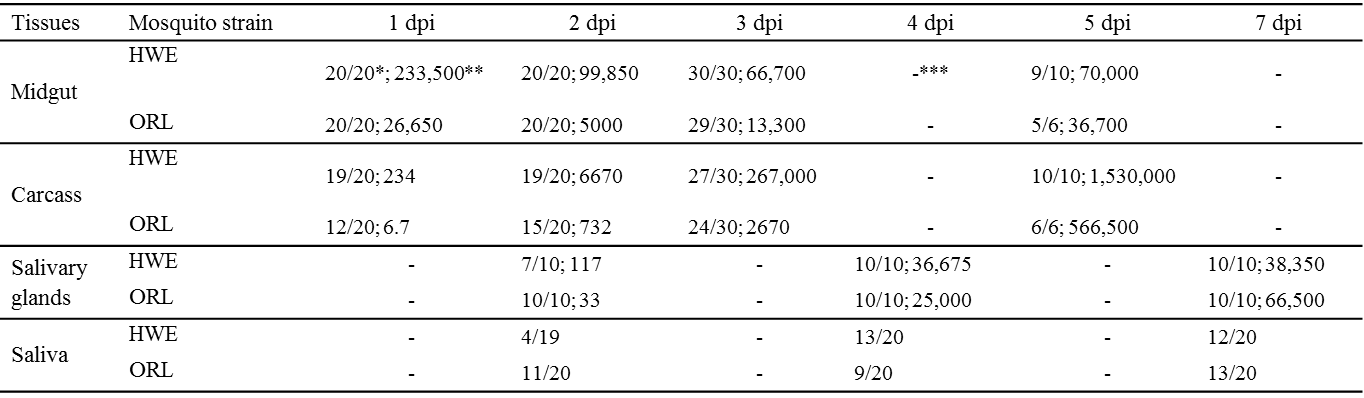


*number of individuals infected with CHIKV / number of individuals challenged with CHIKV; **median CHIKV titre; dpi, days post-infection; *** not analysed.

**Table S2. Primers used for the qRT-PCR detection of apoptosis-related genes.**


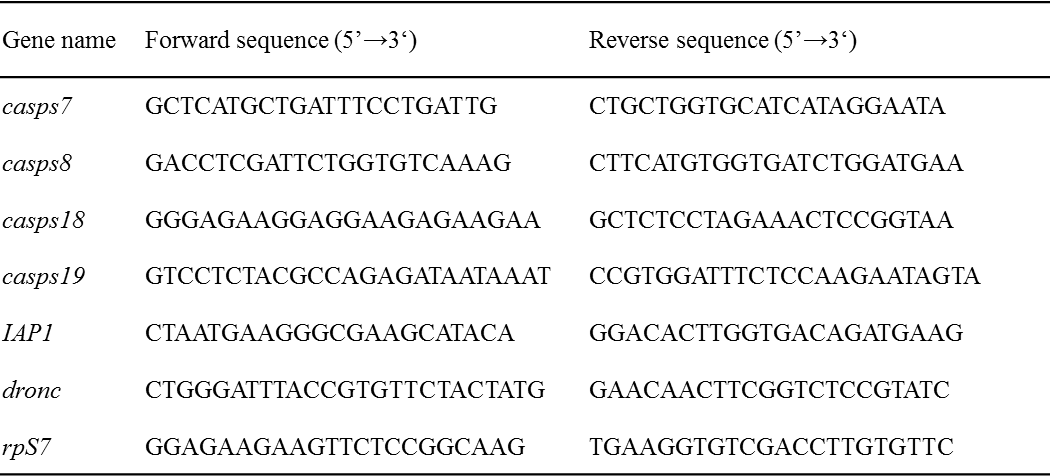


*
